# A CRISPR-Cas9 Assisted Non-Homologous End-Joining Strategy for One-step Engineering of Bacterial Genome

**DOI:** 10.1038/srep37895

**Published:** 2016-11-24

**Authors:** Tianyuan Su, Fapeng Liu, Pengfei Gu, Haiying Jin, Yizhao Chang, Qian Wang, Quanfeng Liang, Qingsheng Qi

**Affiliations:** 1 State Key Laboratory of Microbial Technology, Shandong University, Jinan 250100, People’s Republic of China; 2 National Glycoengineering Center, Shandong University, Jinan, 250100, People’s Republic of China

## Abstract

Homologous recombination-mediated genome engineering has been broadly applied in prokaryotes with high efficiency and accuracy. However, this method is limited in realizing larger-scale genome editing with numerous genes or large DNA fragments because of the relatively complicated procedure for DNA editing template construction. Here, we describe a CRISPR-Cas9 assisted non-homologous end-joining (CA-NHEJ) strategy for the rapid and efficient inactivation of bacterial gene (s) in a homologous recombination-independent manner and without the use of selective marker. Our study show that CA-NHEJ can be used to delete large chromosomal DNA fragments in a single step that does not require homologous DNA template. It is thus a novel and powerful tool for bacterial genomes reducing and possesses the potential for accelerating the genome evolution.

The ability to precisely manipulate the genomes of both eukaryotes and prokaryotes easily and efficiently is highly desirable in applications ranging from genetic analysis of functional genomic loci to metabolic engineering for intentional metabolic flux redistribution^1^,^2^,^3^,^4^. For these purposes, homologous recombination (HR)-based genome engineering has been widely applied^5^,^6^. The introduction of bacteriophage-based recombination proteins revolutionized the process, achieving efficient genetic manipulation especially in prokaryotes^6^,^7^. λ-Red and Rec E/T are two well-known recombinant systems that have been used extensively; however, efficient HR both require a donor DNA fragment flanked by homologous sequences as the editing template^8^. In addition, screening for genetic variations with the desired phenotype requires chromosomal integration of the selective marker (usually, an antibiotic resistance marker) followed by its eventual removal for iterative engineering^8^,^9^.

Novel applications of clustered regulatory interspaced short palindromic repeats (CRISPRs) and CRISPR-associated (Cas) proteins have revolutionized the genomic engineering^10^,^11^,^12^. The CRISPR-Cas9 system, naturally responsible for the adaptive immunity of prokaryotes that is able to recognize and cleave invasive genetic elements, can generate double-stranded breaks (DSBs) at any genomic locus that existing a 5′-NGG-3′ consensus sequence (so called PAM (“protospacer adjacent motif”) sequence) immediately downstream of the target site via the reprogrammable DNA endonuclease activity of Cas9 under the guidance of a engineered single-guide RNA (sgRNA). These DSBs can be repaired either by HR in the presence of the corresponding homologous template or by non-homologous end-joining (NHEJ) in the absence of DNA template accompanied with the modification of target genomic locus^10^,^13^,^14^. In higher organisms, compare with HR, NHEJ is the major DNA repair system to maintain genome stability^15^. NHEJ is perfectly suited for managing DSBs, as this pathway displays no sequence requirements for ligation of DNA ends^16^. However, as broken DNA ends are frequently damaged and need to be modified, the NHEJ repair mechanism tends to be prone to insertion and/or deletion (indel) mutations at the junctional site^17^. Thus, with the assistance of the programmable CRISPR-Cas9 DNA cleavage system, NHEJ can generate frameshift mutations that disrupt the targeted gene without the use of homology repair donor^14^. While this powerful DNA repair mechanism is not prevalent in prokaryotes, the DSB generated by Cas9 is lethal to most microbes due to their general lack of NHEJ pathway^18^,^19^. It is recently shown that the key factors involved in eukaryotic NHEJ, the Ku70/Ku80 heterodimer and DNA ligase IV, exist functional homologs in prokaryotes^20^. Bacterial Ku proteins are much smaller in size than their eukaryotic counterparts but protect damaged DNA by forming ring-like homodimer structures at the ends of the breaks^16^. In certain species, such as *Mycobacterium* and *Bacillus*, the conserved prokaryotic NHEJ pathway safeguards the bacterial genomes against unexpected DSBs and promotes genetic variability^20^,^21^,^22^. This system can also be exploited to develop a bacterial genome engineering approach that is more straightforward than HR-dependent genome engineering, one in which selective marker and the donor DNA template are both unnecessary. Recently, Tong *et al*. have inactivated the target genes using the intrinsic NHEJ pathway in *Streptomyces coelicolor*^23^.

Here, using *Escherichia coli* as example, the DSBs were introduced into chromosome at the desired genomic loci with the help of CRISPR-Cas9 system. We show that these DSBs can be successfully re-joined in the presence of a heterologously expressed NHEJ system with different nucleotides excision, and prove an efficient strategy for larger chromosomal fragments deletion.

## Results

### Design of the CRISPR-Cas9 Assisted Non-Homologous End-Joining System

**Figure 1:**
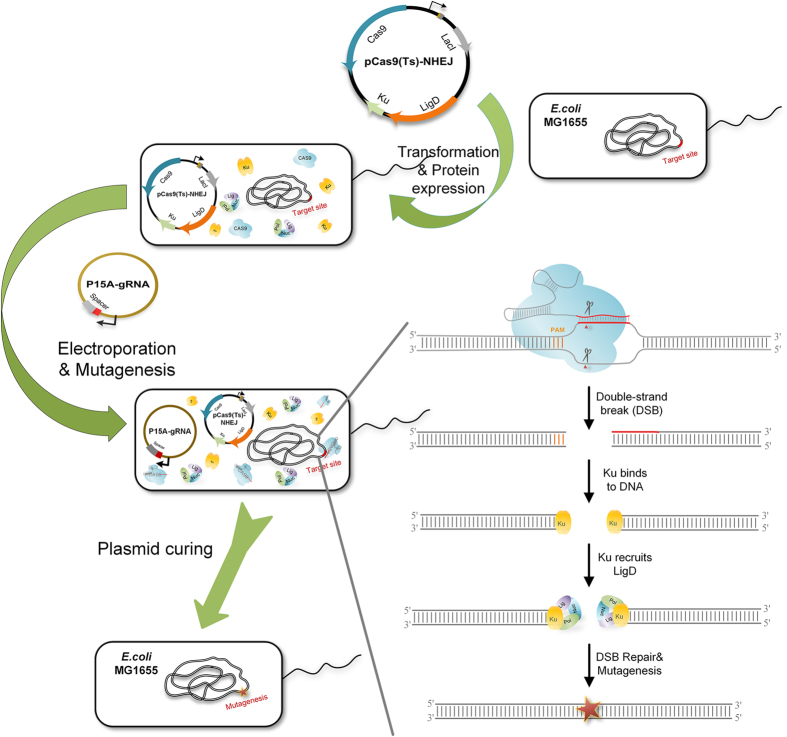
One-step inactivation of chromosomal gene(s) by CRISPR-Cas9 assisted non-homologous end-joining (CA-NHEJ). Cas9 and NHEJ-related proteins (Mt-Ku and Mt-LigD) are expressed in host cells, which are then transformed with a single-guide RNA (sgRNA) donor plasmid to generate double-stranded breaks (DSBs) and trigger indel mutations. Mutagenesis is attributed to the RNA-directed Cas9 cleavage system and the error-prone NHEJ repair system. First, site-specific DSB is generated via sgRNA-directed Cas9 cleavage. The DNA ends are recognized and stabilized by the DNA end-binding protein Mt-Ku. Next, the ATP-dependent DNA ligase Mt-LigD is recruited to the DNA ends; the imprecise repair of DSB results in a frameshift mutation. Finally, only the DSB-repaired colonies lacking the Cas9 targeting site survive CRISPR-Cas9 screening. To further engineer the strain, the sgRNA donor plasmid is cured via an inducible sgRNA-mediated “suicide” strategy, and the temperature-sensitive plasmid pCas9 (Ts)-NHEJ by growing the cells at 42 °C.

The scheme of the CA-NHEJ strategy for one-step gene(s) inactivation is presented in Fig. 1. First, Cas9 from *Streptococcus pyogenes* SF370 and the conserved prokaryotic NHEJ proteins from *Mycobacterium tuberculosis* H37Rv were heterologously expressed in *E. coli*. The sgRNA expression plasmid, containing the information for Cas9 targeting via Watson–Crick base pairing, was then electrotransformed into the host, resulting in the site-specific generation of DSBs and deletion mutations. Briefly, the sgRNA-Cas9 complex binds and cleaves the target DNA strands that generate DSBs at desired genomic loci, which is lethal to wild-type *E. coli*^24^. The imported NHEJ pathway locates and repairs the DSB, such that the cells survive in CRISPR-Cas9 cleavage but carry mutations in the targeted genomic locus. Finally, the helper plasmid is cured through a “suicide” strategy in which an inducible sgRNA targeting the plasmid is expressed and Cas9-mediated cleavage occurs. The overall procedure of gene inactivation by the CA-NHEJ system is remarkably simple—as its only requirements are the construction of a specific sgRNA and single-step electroporation—and thus accelerates the process of genome engineering.

### Demonstration of CA-NHEJ Using Plasmid Containing the *lacZ* Gene

**Figure 2:**
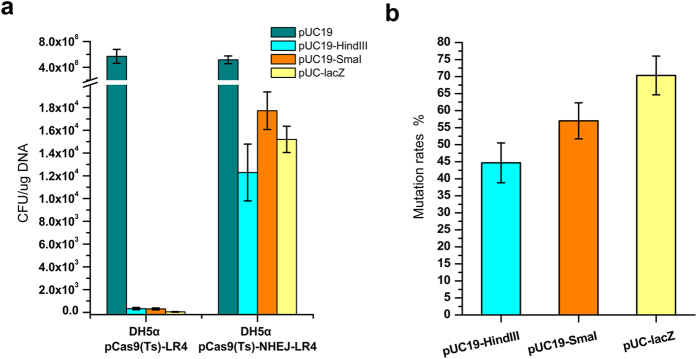
Efficiency and mutation rates of the hetereologous non-homologous end-joining (NHEJ) pathway in *E. coli*. (**a**) Efficiency of the NHEJ system in re-circularizing the *in vitro Hind*III- or *Sma*I-digested pUC19 plasmid and the *in vivo* CRISPR-Cas9 cleaved pUC-lacZ plasmid. The error bars represent standard deviations from three replicate experiments. The results are expressed as colony-forming units (CFU) per μg of plasmid DNA. *mku and ligd*, derived from *M. tuberculosis* H37Rv and involved in the NHEJ pathway, are hetereologously expressed in *E. coli* using a strong constitutive P_J23119_ promoter. (**b**) The mutation rates of the NHEJ system with different artificially created DSBs. The mutation rates are statistically determined based on the proportion of white colonies on the X-gal plate. The error bars represent standard deviations from three replicate experiments.

As proof of concept, a plasmid-based gene inactivation experiment was carried out using CA-NHEJ. In the first step, the sgRNA targeting the *lacZ* gene on plasmid pUC-lacZ was used together with the CRISPR-Cas9 system to investigate the DSB generation. Plasmid pUC-lacZ was electrotransformed into *E. coli* DH5α (*ΔlacZ)* containing the complete CRISPR-Cas9 system targeting the alpha-fragment of *lacZ* in parallel with plasmid pUC19, which served as the negative control that without sgRNA target site. The result revealed 1.3 × 10^7^ fold decrease of transformation efficiency in pUC-lacZ than that of pUC19, which demonstrated the high efficiency of the CRISPR-Cas9 system and the weak capability of endogenous DSB repair in wild-type *E. coli* (Fig. 2a). The utility of the NHEJ system was then demonstrated by re-circularization of the restriction endonuclease-linearized plasmid^25^. A 5′-overhang or blunt end of the DSB in the alpha-fragment of *lacZ* of plasmid pUC19 was generated by *Hin*dIII or *Sma*I digestion, respectively. Transformants harboring the re-circularized plasmid were easily identified by blue-white screening (see Supplementary Fig. 1). The amount of transformant colonies increased by several dozens of times than that of control, indicating efficient DNA repair of the hetereologous NHEJ mechanism in *E. coli* (Fig. 2a). Blunt end-joining was slightly more efficient than sticky end-joining, which probably reflected the terminal sequence specificity of the various DSBs. Statistical analysis of the proportion of white colonies demonstrated a low fidelity of the heterologous NHEJ DNA repair system in *E. coli* (Fig. 2b and Supplementary Fig. 1). Moreover, DSB with sticky end (generated by *Hin*dIII digestion) was repaired with slightly greater accuracy than that with blunt end (generated by *Sma*I digestion) (55.3 ± 4.4% vs. 43.0 ± 4.0%) (Fig. 2b).

*In vivo* DSB generation using the CRISPR-Cas9 system followed by NHEJ repair was confirmed by transforming plasmid pUC-lacZ into a host harboring both systems, which therefore exhibited an increased transformation efficiency and high *lacZ* mutation ratio (Fig. 2a and Supplementary Fig. 1). The transformation efficiency was 428 ± 81 fold higher in this strain than in the NHEJ-deficient strain, while for the DSB generated by Cas9 *in vivo*, the fidelity of repair was much lower than the restriction endonuclease-linearized plasmid *in vitro* (Fig. 2b). Only 29.7 ± 4.2% of the colonies retained the *lacZ*^*+*^ phenotype after DSB repair.

Sanger sequencing was used to determine the exact mutation patterns at the junctional site (Supplementary Fig. 2b). The deleted DNA fragments ranged from 10 to 267 bp in length, and there was no directional bias regarding the end nucleotide deletions. Interestingly, we found that the micro-homology-dependent terminal re-ligation occurred at the DSB. These results suggested that the intracellular exonucleases and the nuclease activity of Mt-LigD contributed to process the DNA ending at DSB, prior to joining, and that micro-homology flanking by the DNA junction improved end-joining.

### Targeting of Gene on the Chromosome

**Figure 3:**
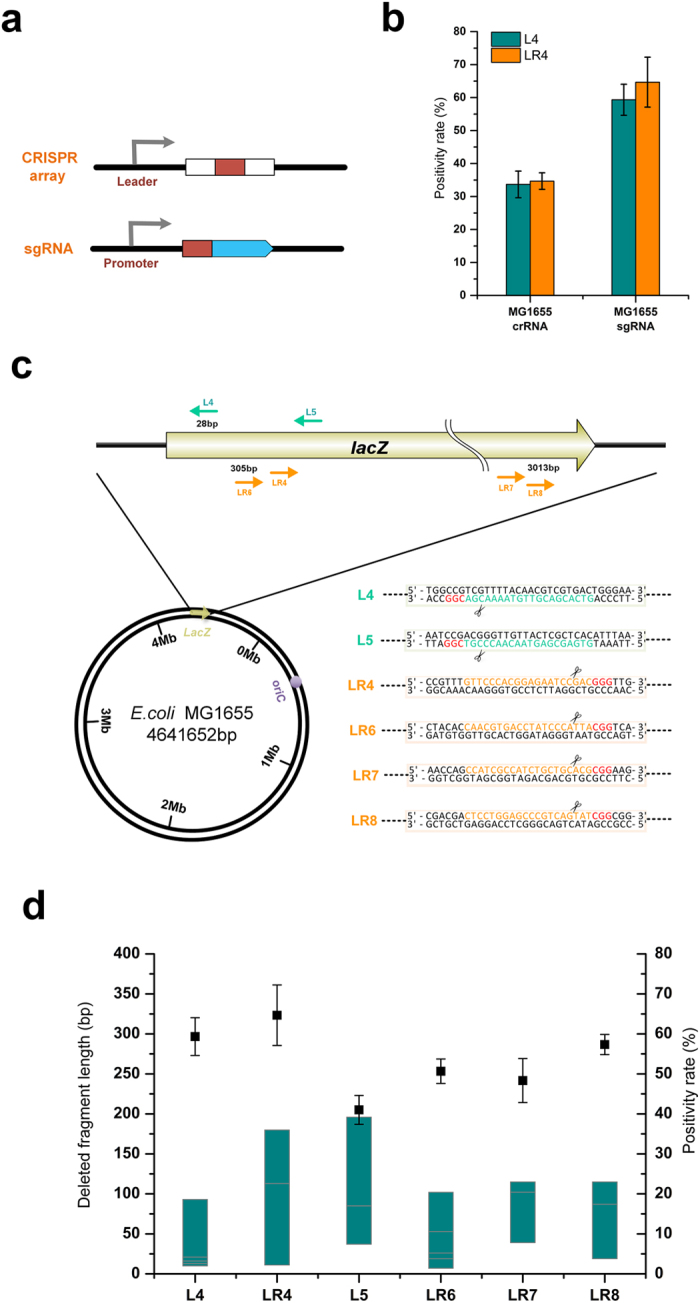
Gene disruption using CA-NHEJ in *E. coli.* (**a**) The structure of the natural CRISPR array and the artificial single-guide RNA (sgRNA). White: repeat sequences; red: spacer sequences; blue: RNA domains for Cas9 binding and a 40-nt transcription terminator. (**b**) The *lacZ* mutagenesis positivity rate in strain MG1655 using spacers expressed by the CRISPR array or sgRNA cassette. Two spacers, L4 and LR4, targeting the sense strand and the antisense strand of *lacZ*, were tested, respectively. The positivity rate was statistically calculated based on the proportion of white colonies on the X-gal plate. The error bars represent standard deviations from three replicate experiments. (**c**) Distribution of sgRNAs designed to target the *lacZ* gene in strain MG1655. The location of the designed spacers, the spacer sequences, the PAM (protospacer adjacent motif) sequences, and the cleavage sites are detailed and highlighted. (**d**) The positivity rate of *lacZ* mutagenesis and the range of DNA end fragment deletion using various sgRNAs. The positivity rate shown is representative of three replicate experiments. Solid square: the positivity rate of *lacZ* mutagenesis; dark cyan bar: the length of DNA end fragment deletion. For each sgRNA target, eight white colonies were randomly picked for Sanger sequencing to determine the length of the deleted fragment at the junction.

To investigate the ability of the CA-NHEJ system to disrupt gene on the chromosome, *E. coli* strain MG1655 was selected as the target strain. The CRISPR donor plasmid harboring the LR4 CRISPR array targeting *lacZ* was electrotransformed into strain MG1655 containing pCas9 (Ts)-NHEJ; the genotype of the *lacZ* gene in the resulting strain was identified through blue-white selection. As shown in Supplementary Fig. 3, the presence of several white colonies on the X-gal plate confirmed chromosomal gene disruption by the CA-NHEJ system. However, most colonies were still *lacZ*^+^ and the positivity rate of the initial CA-NHEJ system was only 34.6 ± 2.5% (Fig. 3b).

To determine why these colonies escaped CRISPR-mediated cleavage, the non-mutated colonies were sequenced (Supplementary Fig. 4). In eight of the 10 *lacZ*^+^ colonies, recombination between two repeat sequences of the CRISPR array occurred that caused the targeting spacer sequence eliminated. This phenomenon was described in a previous report^10^. To prevent HR, the sgRNA donor plasmid p15A-gRNA, driving expression of the spacer, was used to induce the CRISPR-Cas9 system, thus circumventing the occurrence of repeat sequences construction^26^ (Fig. 3a). With this modifications, the efficiency of *lacZ* inactivation improved considerably, 64.5 ± 7.5% of the cells acquired a *lacZ*- phenotype (Fig. 3b). Similar results were obtained for another target site, L4 (Supplementary Fig. 3). These results demonstrated that sgRNA construction are effective in avoiding HR (Fig. 3b).

The effects of various targeting sites were then investigated (Fig. 3c). Six spacers distributed on the *lacZ* gene were designed according to the previously described principles^27^,^28^. Briefly, polyT (>4 T’s) sequences were avoided in spacer construction to prevent unexpected translational termination. The GC content of the spacers was controlled such that it remained within the range of 30–80%. Whole-genome BLAST was used to preclude undesired “off-targets”. Two spacers, L4 and L5, were designed on the sense strand and four others targeted the antisense strand. All six spacers effectively inactivated *lacZ*, demonstrating the universality of the CA-NHEJ system. The efficiency of mutagenesis using different spacers was between 41.0 ± 3.6% and 64.5 ± 7.5%, depending on the terminal sequence specificity of the various DSBs (Fig. 3d). Regardless of whether the sense or the antisense strand was targeted, mutagenesis was effectively triggered, which confirmed that the system does not exhibit DNA strand preference. The excision of several to hundreds of nucleotides at the junctional site was detected (Fig. 3d). The range of DNA end deletions by different sgRNAs was relatively consistent at around 200 bp, a size smaller than that of most genes.

To cure the plasmids involved in the CA-NHEJ system, an inducible sgRNA cassette targeting the p15A replicon was cloned into pCas9 (Ts)-NHEJ, resulting in pcurCas9 (Ts)-NHEJ. The p15A-gRNA plasmid was eliminated via inducible cleavage by Cas9, and plasmid pcurCas9 (Ts)-NHEJ itself was cured by incubation at 42 °C.

### The Deletion of Large Chromosomal DNA Fragments in Bacterial Genomes

To delete large DNA fragments from the bacterial chromosome, sgRNA pairs were used to generate double DSBs simultaneously. The DNA fragment located between the two DSBs would be “cut off” from the chromosome, along with re-ligation of the genomic endpoints of the DSBs. To improve the efficiency of the CA-NHEJ system in the deletion of large fragments, *cas9, mku, and ligd* were constructed on a high-copy-number plasmid, resulting in pwtCas9-NHEJ. In addition, a one-step sgRNA pair assembly method was designed to simplify construction of the sgRNA pairs (see Supplementary Fig. 5).

**Figure 4:**
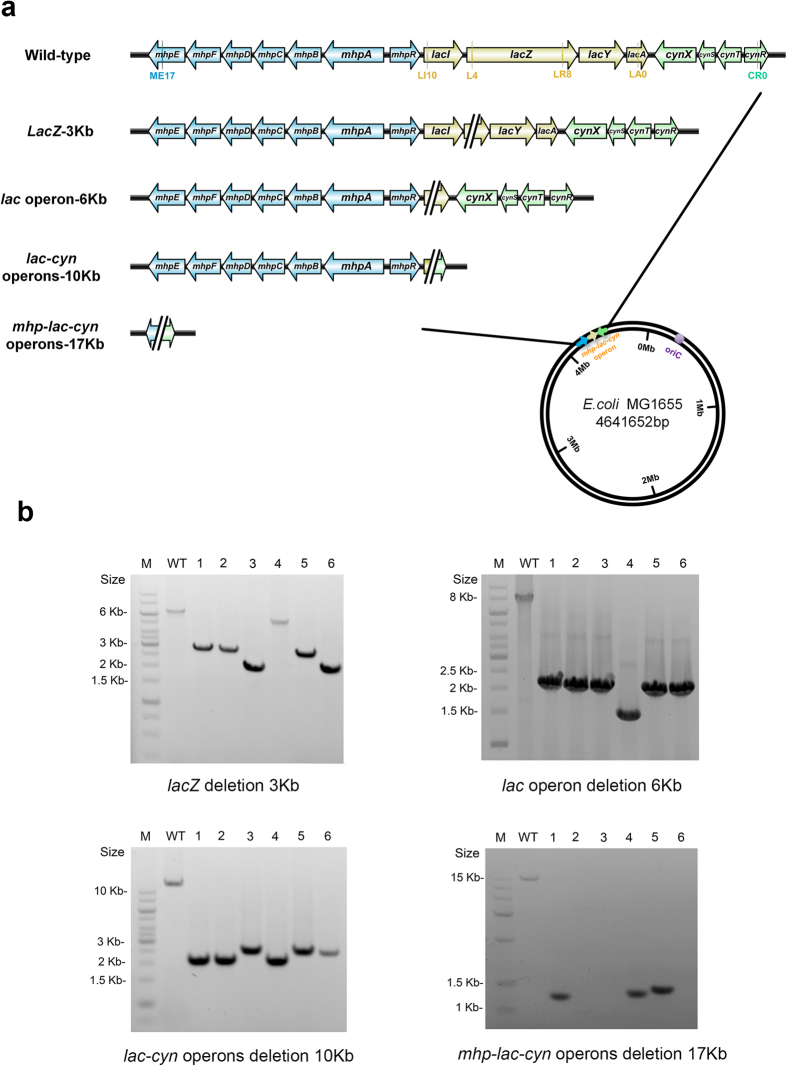
Large DNA fragment deletion in the *E. coli* MG1655 chromosome by CA-NHEJ. (**a**) Schematic of chromosomal fragment deletions using CA-NHEJ. Four sgRNA pairs, L4&LR8, LI10&LA0, LI10&CR0, ME17&CR0, were designed for the deletion of the *lacZ* gene, *lac* operon, *lac-cyn* operons, and *mhp-lac-cyn* operons, respectively. The distribution of the sgRNA pairs and the DNA fragments expected to be deleted by separate sgRNA pairs are detailed and highlighted. Cyan: the *mhp* operon; tan: the *lac* operon; green: the *cyn* operon. (**b**) PCR analysis of six transformants from each engineering experiment, used to identify the lengths of deleted DNA fragments by various sgRNA pairs.

As shown in Fig. 4a, four sgRNA pairs were designed to target genomic DNA fragments ranging from 3 to 17 kb. To facilitate the screening of positive colonies, all of the targeted fragment contained *lacZ.* The sgRNA pairs L4&LR8, LI10&LA0, LI10&CR0, and ME17&CR0 were cloned into plasmid p15A-BsaI-BbsI-gRNA. The resulting constructs were then separately electrotransformed into strain MG1655 containing pwtCas9-NHEJ. Although DNA fragment deletions were found in all cases (Fig. 4b), fewer colonies were obtained for deletions >10 kb. The efficiency and positivity rate were correlated negatively with the length of the deleted DNA fragments (Table 1). Dual sgRNAs was more toxic to the cells relatively to the single sgRNA based on the number of viable CFUs (Table 1). Furthermore, for the 3 kb *lacZ* gene deletion, whether the mutation was the result of a frameshift mutation or the complete deletion of the *lacZ* gene could not be determined by blue-white selection. To exclude false-positive colonies, that is, those with *lacZ* frameshift mutations, the detailed mutation patterns of 46 randomly selected white colonies were determined by PCR. In 30 colonies, the fragment deletions were around 3 kb; thus, in 65.2% (30/46) of the white transformant colonies, the *lacZ* gene was deleted completely (Supplementary Fig. 6). The size of the PCR products in the remaining colonies was approximately the same as the wild-type product, which implied that only a *lacZ* frameshift mutation had occurred. Therefore, the final positivity rate for the complete deletion of *lacZ* was calculated by proportionally deducting the number of false-positive colonies carrying the frameshift mutation.

On gel electrophoresis, several PCR products were smaller than expected, indicating that the lengths of the deleted fragments in certain colonies were accordingly longer (Fig. 4b). Moreover, fragment deletions >19 kb were obtained using the sgRNA pair ME17&CR0, as confirmed by the lack of PCR products using primers MLC-JF/MLC-JR (Fig. 4b). These results indicated the occurrence in the CA-NHEJ system of excess resection of the DNA ends before double DSBs repair.

## Discussion

The novel strategy for HR-independent genome editing of prokaryotic cells presented in this study is the first reported *E. coli* engineering method employing a hetereologous NHEJ mechanism. Only a specific sgRNA construction and single-step electroporation are needed to generate DSBs and mutagenesis, which simplifies and accelerates prokaryotic genome modification considerably. The sgRNA plasmid involved in the CA-NHEJ system is cured using an inducible sgRNA cassette targeting the p15A replicon, which further simplifies the genetic manipulation of the host.

The long-standing limitations of traditional, HR-dependent genetic engineering methods have been how to simplify the operational procedure and realize complicated genomic manipulation^1^,^5^. Various improved genome engineering methods based on phage recombination systems were developed; but while they facilitated the reprogramming of complex biological processes and accelerated the advance of metabolic engineering and synthetic biology, all of them required the construction of a specific homologous DNA editing template, usually also containing a selective marker, for each genomic locus^1^,^29^,^30^,^31^. This resulted in a labor-intensive and time-consuming procedure, especially for large-scale multiplex or iterative whole-genome engineering^9^ (Table 2). The use of short single-stranded DNA (ssDNA) as templates for recombineering that is independent of the selective marker greatly facilitated whole-genome engineering^32^, as exemplified by multiplex automated genome engineering (MAGE)^33^,^34^. However, the genetic modification range of ssDNA recombineering is constrained by the length of the ssDNA, and large-scale chromosomal DNA fragment deletion is therefore not possible using that method. Several strategies have been proposed for the deletion of large DNA fragments on bacterial chromosomes, but they generally rely on the previous introduction of exogenous recombinase or meganuclease sites into the bacterial chromosome at a specific genomic locus, followed by a laborious two-step procedure for chromosomal rearrangement^35^,^36^.

The CRISPR-Ca9 system has been broadly adopted in markerless genome engineering, and its utility and flexibility in genetic manipulation of the bacterial genome have improved dramatically^34^,^37^,^38^,^39^,^40^,^41^,^42^. Recently, Beier *et al*. described a CRISPR-guided nickase system that can be programmed to induce recombination events at targeted genomic loci, leading to the deletion of large fragments on the *E. coli* chromosome^43^. Nevertheless, the appropriate repetitive sequences in close proximity to the targeted genomic locus are required to promote HR. The CA-NHEJ strategy described here takes advantage of the error-prone NHEJ DNA repair mechanism and the CRISPR-Cas9 system for bacterial genome engineering. Through the efficient site-specific DNA cleavage and template-independent DSB repair, genome modification can be achieved effectively and rapidly in the absence of a donor DNA editing template and selective marker (Table 2). The efficient disruption of *lacZ* by various target sites, as shown in this study, confirmed the high reliability of this method for engineering any genomic locus. Because it is HR-independent, CA-NHEJ is highly suitable for the large-scale deletion of chromosomal DNA fragments and therefore of tremendous value in functional genomics research involving large numbers of genes and whole-genome engineering (Table 2). During the preparation of this manuscript, Cui *et al*. have also tried to rescue the *E. coli* from Cas9-mediated the break of genome DNA using the NHEJ pathway^44^. However, they found the introduction of the NHEJ system in the chromosome of *E. coli* strain N4278 can repair the DSB generated by Cas9 but with an efficiency lower than the frequency of background mutations of the CRISPR system that, so far, was unable to employ in gene inactivation. The DSB on the chromosome is highly toxic to cells, so efficiently and timely repair of the genomic damage is crucial to enable the cell survive from Cas9 cleavage. We suggest that the inadequate expression of the NHEJ enzymes in chromosome with single copy is possibly the main reason for inefficiently repairing DSBs, which interrupts the application of NHEJ in genome editing at genomic locus of interest^45^. Xu *et al*. also failed to edit *Clostridium cellulolyticum* genome via NHEJ on account of the low expression of the intrinsic NHEJ enzymes that further confirmed our conclusion^46^. Therefore, a high-copy plasmid pwtCas9-NHEJ and the strong constitutive P_J23119_ promoter were used in this work to express NHEJ enzymes in high activity and effective gene inactivation or large chromosomal fragment deletion was achieved using this strategy. In addition, the use of dual crRNA and tracrRNA system may also cause possible recombination between the two repeat sequences that increasing the background mutations of CRISPR system^10^. We solved this issue by using customized sgRNA as the spacer donor to avoid the repeat sequences construction.

In bacteria, yeast, and human cells, complicated molecular mechanisms of stress-induced mutagenesis have evolved to improve the genetic diversity of the respective populations^47^,^48^. DSB-dependent stress-induced mutations in bacteria serve as a general driving force for both genome evolution and the adaptation to environmental challenges, and therefore is a powerful tool for evolutionary engineering^49^. However, the low frequency and the high degree of randomness of this stress-induced mutagenesis (SIM) in *E. coli* greatly restrict its extensively application. By contrast, in the CA-NHEJ strategy described herein, the CRISPR-Cas9 system possesses the potential to generate the desired DSBs at whole-genome scale, thus allowing rational or semi-rational directed evolution of the bacterial genome to generate the phenotypes of interest and to understand the nature of evolution.

## Materials and Methods

### Strains and Culture Conditions

The *E. coli* K-12 strain DH5α was used as the host strain for molecular cloning and manipulation of plasmids. *E. coli* strains DH5α (Δ*lacZ*) was obtained by the one-step gene inactivation method described previously^8^. Primers used for gene knockout are listed in Supplementary Table 1. *E. coli* strains DH5α, DH5α (Δ*lacZ*) and MG1655 were employed as the host for genetic engineering using CA-NHEJ.

Strains for cloning were grown in Luria-Bertani (LB) medium (10 g/l tryptone, 5 g/l yeast extract, and 10 g/l NaCl) supplemented with appropriate antibiotic (ampicillin (100 mg/l), kanamycin (25 mg/l), chloramphenicol (25 mg/l), and spectinomycin (50 mg/l)). When appropriate, anhydrotetracycline (aTc), 5-bromo, 4-chloro-3-indolyl-beta-D-galactopyranoside (X-gal) and isopropyl β-D-1-thiogalactopyranoside (IPTG) were supplemented into the cultures as followings: aTc (1 μM), X-gal (40 mg/l), IPTG (0.2 mM).

### Plasmid Construction

All plasmids and primers used in this work are listed in Supplementary Tables 1 and 2. The sequences of core elements in this study was described in Supplementary Note 1. To generate pUC-lacZ, the *lacZ* gene was obtained using lacZ-F/lacZ-R as the primers and the genome of *E. coli* strain MG1655 as the template. Plasmid pUC-lacZ was constructed by ligating the *Hin*dIII*/Xba*I double-digested *lacZ* gene into pUC19. To construct pCas9 (Ts), two DNA fragments, the psc101-*Cm* module and *cas9*, were amplified using Cm-Ori-F/Cm-Ori-R and cas9-F/cas9-R as the primers and the plasmids pCP20 and pCas9 (Addgene plasmid # 42876) as the templates, respectively. These two fragments, with 30–40 homologous bases, were assembled *in vitro* by Gibson assembly^50^. The genome of *M. tuberculosis* H37Rv (NC_000962.3) was then used as the template to amplify the conserved NHEJ pathway gene cassettes P_J23119_*-ligd* and *mku* by primers 23119-ligd-F/23119-ligd-R and mku-F/mku-R, respectively. Fusing the p23119*-ligd* fragment and the *mku* gene *in vitro* using splice overlap extension-PCR yielded p23119*-ligd*-*mku*, which was then subcloned into the *Bgl*II and *Nsi*I sites of pCas9 (Ts), under the control of constitutive P_J23119_ promoter, generating pCas9 (Ts)-NHEJ. The LR4-CRISPR array module, obtained using CRISPR-F/CRISPR-R as the primers and pCRISPR-LR4 as the template, was then digested with *Xba*I. The resulting fragment was ligated into pCas9 (Ts) and pCas9 (Ts)-NHEJ to generate pCas9 (Ts)-LR4 and pCas9 (Ts)-NHEJ-LR4, respectively. To construct pcurCas9 (Ts)-NHEJ, the *lacI* gene and trc-p15A-sgRNA fragment with 30 bp of homologous bases was synthesized (Genscript) and assembled with *Xba*I-digested pCas9 (Ts)-NHEJ.

To express the NHEJ pathway, the P_J23119_-*ligd-mku* expression cassette was amplified using the primers NHEJ-F/NHEJ-R and the plasmid pCas9 (Ts)-NHEJ as the template. The resulting fragment was then subcloned into the *Spe*I site of pwtCas9-bacteria (Addgene plasmid # 44250), generating pwtCas9-NHEJ. To obtain the sgRNA expression plasmid p15A-gRNA, the *Bsa*I-sgRNA module containing two *Bsa*I type IIS sites, allowing the insertion of new spacers, was synthesized by Genscript. The *Bsa*I-sgRNA module, amplified using the primers sgRNA-F/sgRNA-R with 30–40 bp of homologous bases, was then assembled with the spectinomycin resistance cassette and a p15A replicon fragment, generated by the amplification of pTKRED using the primers Spc-F/Spc-R and pCas9 using the primers P15A-F/P15A-R. To construct p15A-BsaI-BbsI-sgRNA, the *Bbs*I-sgRNA-terminator module containing two *Bbs*I type IIS sites was synthesized by Genscript. This module was then digested with *Bam*HI and cloned into the *Bam*HI-digested p15A-gRNA plasmid, generating p15A-BsaI-BbsI-sgRNA.

### Design and Construction of the CRISPR Target

The CRISPR target sequences used in this work are listed in Supplementary Table 3. The golden gate cloning method was used to generate specific CRISPR array containing the new 30 bp spacer sequence in plasmid pCRISPR (Addgene plasmid # 42875) and sgRNA cassette containing the new 20 bp spacer sequence in plasmid p15A-gRNA as previously described^10^. Briefly, the two newly synthesized complementary oligos were annealed in oligo DNA annealing buffer (10 mM Tris pH 8.0, 50 mM NaCl, 1 mM EDTA) to obtain the short double-stranded spacer sequences. A 20-μL reaction system, containing 300 ng of donor plasmid, 10 pmol of annealed spacer DNA, 10 U of *Bsa*I, 10 U of T4 polynucleotide kinase, and 200 U of T4 DNA ligase (all three enzymes from New England Biolabs) in T4 DNA ligase buffer, were carried out for 10 cycles at 37 °C/15 min and 20 °C/25 min followed by an 80 °C/10 min inactivation step. The ligation product was directly transformed into *E. coli* DH5α for further verification and manipulation.

To simplify the generation of specific sgRNA pair cassettes in plasmid p15A-BsaI-BbsI-sgRNA, a one-step digestion-ligation method based on golden gate cloning was developed (Supplementary Fig. 5). Thus, sgRNA pair was cloned into p15A-BsaI-BbsI-sgRNA by two rounds of iterative golden gate cloning in a 30-μL reaction system. Briefly, the procedure used in the first round was the same as described above for single-spacer construction. After 10 cycles of enzymatic digestion and ligation, another annealed spacer DNA and 10 U of type IIS restriction endonuclease *Bbs*I (New England Biolabs) were added to the reaction system, followed by another 10 cycles of digestion-ligation to ligate the second spacer. The inactivated ligation product was treated with the Plasmid Safe Exonuclease (Epicenter) for 30 min at 37 °C to remove the un-ligated plasmid fragment and then directly transformed into *E. coli* DH5α.

### Verification of the CRISPR and NHEJ System

*E. coli* DH5α (Δ*lacZ*) containing pCas9 (Ts)-LR4 expressing the complete CRISPR-Cas9 system targeting the alpha-fragment of the *lacZ* gene was selected as the host strain to verify the CRISPR cleavage system. The strain was pre-cultured in 5 mL of LB medium at 30 °C overnight. The overnight culture was diluted 200-fold and grown at 30 °C to an OD_600nm_ of ~0.6. The cells were harvested at 4 °C and used to transform plasmid pUC-lacZ by electroporation. The electroporated cells were immediately re-suspended in 1 mL of LB medium and then allowed to recover at 30 °C for 1 h, after which they were plated on LB agar with appropriate antibiotic.

*E. coli* DH5α containing pCas9 (Ts)-NHEJ-L4 expressing the complete NHEJ pathway was chosen as the host strain to characterize the NHEJ system. Plasmid pUC19 was linearized *in vitro* using *Hin*dIII or *Sma*I, respectively. The linearized plasmid fragments were separately electrotransformed into DH5α containing pCas9 (Ts)-NHEJ-L4 according to a standard electroporation protocol^10^. After cultivation, the cells were plated on LB agar with X-gal, IPTG, and appropriate antibiotic. The bacterial colonies grown on the plates were counted and analyzed using an automatic colony counters (Shineso).

To verify the CA-NHEJ system, *E. coli* DH5α (Δ*lacZ)* cells containing pCas9 (Ts)-NHEJ-L4, expressing the complete CRISPR-Cas9 system targeting the alpha-fragment of *lacZ* and the NHEJ pathway, were selected as the host strain. Briefly, the pUC-lacZ plasmid was used to transform strain DH5α (*ΔlacZ)* containing pCas9 (Ts)-NHEJ-L4 by electroporation. The resulting colonies were counted and analysed as described above.

### Genome Engineering Using CA-NHEJ

To inactivate *lacZ* on the *E. coli* chromosome using CA-NHEJ, strain MG1655 was first transformed with the temperature-sensitive plasmid pCas9 (Ts)-NHEJ containing *cas9* and P_J23119_-*mku-ligd*. A single colony obtained from the plate was pre-cultured in 5 mL of LB medium at 30 °C overnight. One milliliter of the overnight culture was inoculated into 50 mL of LB medium and grown at 30 °C with shaking (220 rpm) until an OD_600nm_ of 0.4–0.6 was reached (~2 h). The cells were harvested at 4 °C and washed according to the electroporation protocol. Plasmid pCRISPR, expressing the specific CRISPR target, was used to transform the cells by electroporation, thus allowing the generation of DSBs. One milliliter of room temperature LB medium was immediately added to the cells, which were then allowed to recover at 30 °C for 1–2 h, followed by plating on LB agar with X-gal, IPTG, and the appropriate antibiotic. The transformants were grown on the plates and the colonies were counted and analyzed as described above.

To avoid possible HR in the CA-NHEJ system, plasmid p15A-gRNA was tested as the CRISPR target donor. The procedures of the improved CA-NHEJ system was consistent with the protocol described above.

To cure the plasmids involved in the CA-NHEJ system, an inducible p15AsgRNA cassette targeting the p15A replicon of p15A-gRNA was cloned into pCas9 (Ts)-NHEJ, generating pCas9 (Ts)-NHEJ-sgRNA. After the host strain genome was modified using pCas9 (Ts)-NHEJ-sgRNA, IPTG was added to the culture to induce the expression of the p15AsgRNA cassette, resulting in the elimination of the p15A-gRNA plasmid through Cas9 mediated cleavage. To cure the temperature-sensitive pCas9 (Ts)-NHEJ plasmid, the cells were then grown at 42 °C.

To delete large genomic fragments using the CA-NHEJ system, p15A-BsaI-BbsI-sgRNA, containing the sgRNA pairs L4&LR8, LI10&LA0, LI10&CR0, or ME17&CR0, was used as the individual CRISPR target donor to allow the simultaneous generation of double DSBs.

### Mutations Analysis

The mutations were confirmed by polymerase chain reaction (PCR) analysis and Sanger sequencing. The primers used to detect the mutations are listed in Supplementary Table 1.

To analyze the fragment deleted by sgRNA pairs L4&LR8, LI10&LA0, LI10&CR0, and ME17&CR0, lacZ-JF/lacZ-JR, Lac-JF/Lac-JR, LC-JF/LC-JR, and MLC-JF/MLC-JR, flanking the endpoints of the respective targeted fragment, were used as the primers and the genome of the resulting strains as templates for PCR analyses. The *E. coli* genome was extracted using the TIANamp bacterial DNA kit (Tiangen). PCR was carried out using a Life-Touch thermocycler (BIOER) and *LA Taq*™ version 2.0 Plus dye DNA polymerase (Takara). The PCR products were visualized by 0.8% agarose gel electrophoresis. Sanger sequencing to precisely determine the pattern of mutations was performed by Biosune.

## Additional Information

**How to cite this article**: Su, T. *et al*. A CRISPR-Cas9 Assisted Non-Homologous End-Joining Strategy for One-step Engineering of Bacterial Genome. *Sci. Rep.* **6**, 37895; doi: 10.1038/srep37895 (2016).

**Publisher's note:** Springer Nature remains neutral with regard to jurisdictional claims in published maps and institutional affiliations.

## Supplementary Material

Supplementary Information

## Figures and Tables

**Table 1:** Efficiency and positivity rate of various size chromosomal fragment deletions by CA-NHEJ.

sgRNA pairs	Length of deleted fragments	Number of white colonies	Number of total colonies	Average positivity rate (%)
L4	~100 bp	521 ± 75	882 ± 133	59.3 ± 4.5
L4&LR8	~3 Kb	83 ± 14	112 ± 12	49.1 ± 2.1*
LI10&LA0	~6 Kb	28 ± 7	74 ± 5	36.8 ± 6.5
LI10&CR0	~10 kb	12 ± 3	51 ± 13	25.6 ± 4.0
ME17&CR0	~17 kb	4 ± 1	24 ± 3	17.3 ± 3.6

^*^The proportion of white colonies was 75.3 ± 3.2% on the blue-white screening plate, while 65.2% (30/46) white colonies possessed complete deletion of *lacZ* according PCR analysis (Supplementary Fig. 6). Hence, the final positivity rate for deletion of the complete *lacZ* gene was corrected by deducting the false-positive colonies (65.2% × 75.3 ± 3.2%). All results were obtained from three independent trials.

**Table 2:** Comparison of genome engineering achieved with homologous recombination (HR) and non-homologous end-joining (NHEJ).

Genome engineering		Screening method	Operational complexity	Large fragment deletion	Genome-scale engineering	References
Principle	Method					
NHEJ	CA-NHEJ	Not required	Easy	Available	Efficient	This study
HR	CRISPR-Cas9 assisted HR	Not required	Strenuous	Available	Difficult	10, 29, 43
HR	Double-stranded DNA recombineering	Required	Strenuous	Time consuming	Difficult	8, 9
HR	Single-stranded DNA recombineering	Not required	Easy	Incapable	Efficient	32, 33
