# Supplementary material for: A CRISPR-Cas9 Assisted Non-Homologous End-Joining Strategy for One-step Engineering of Bacterial Genome
Source: Sci Rep. 2016 Nov 24;6:37895. doi: 10.1038/srep37895 (PMC5121644; doi:10.1038/srep37895)
Supplement: Supplementary Information [file srep37895-s1.pdf]

SUPPLEMENTARY INFORMATION

A CRISPR-Cas9 Assisted Non-Homologous End-Joining Strategy for One-step Engineering of Bacterial Genome

Tianyuan Su[1], Fapeng Liu[1], Pengfei Gu[1], Haiying Jin[1], Yizhao Chang[1], Qian Wang[2], Quanfeng Liang[1], Qingsheng Qi[1, 2]

[1]State Key Laboratory of Microbial Technology, Shandong University, Jinan 250100, People's Republic of China

[2]National Glycoengineering Center, Shandong University, Jinan, 250100, People's Republic of China

* Corresponding author: Qingsheng Qi

E.mail: qiqingsheng@sdu.edu.cn

Tel: +86-531-88365628;

Fax: +86-531-88362897

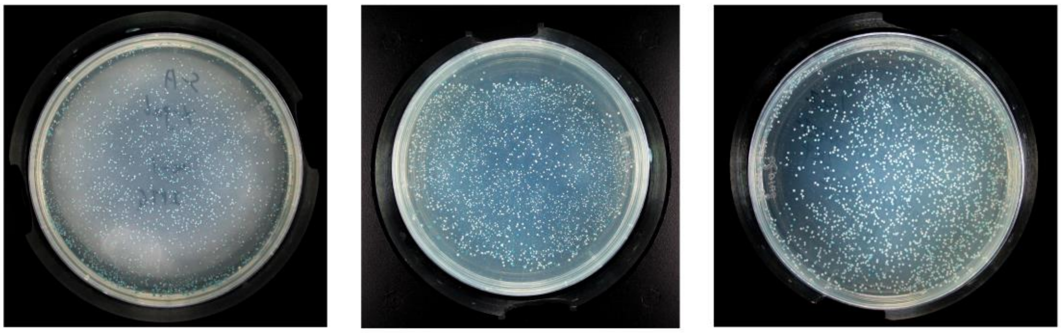

HindIII-linearized pUC19 *SmaI*-linearized pUC19 CRISPR-cleaved pUC-lacZ

Supplementary Figure 1. Blue-white screening of the transformants to determine the efficiency and fidelity of the heterogenous NHEJ pathway in *E. coli*. White colonies represent the *lacZ*$^{-}$ genotype and blue colonies represent the *lacZ*$^{+}$ genotype.

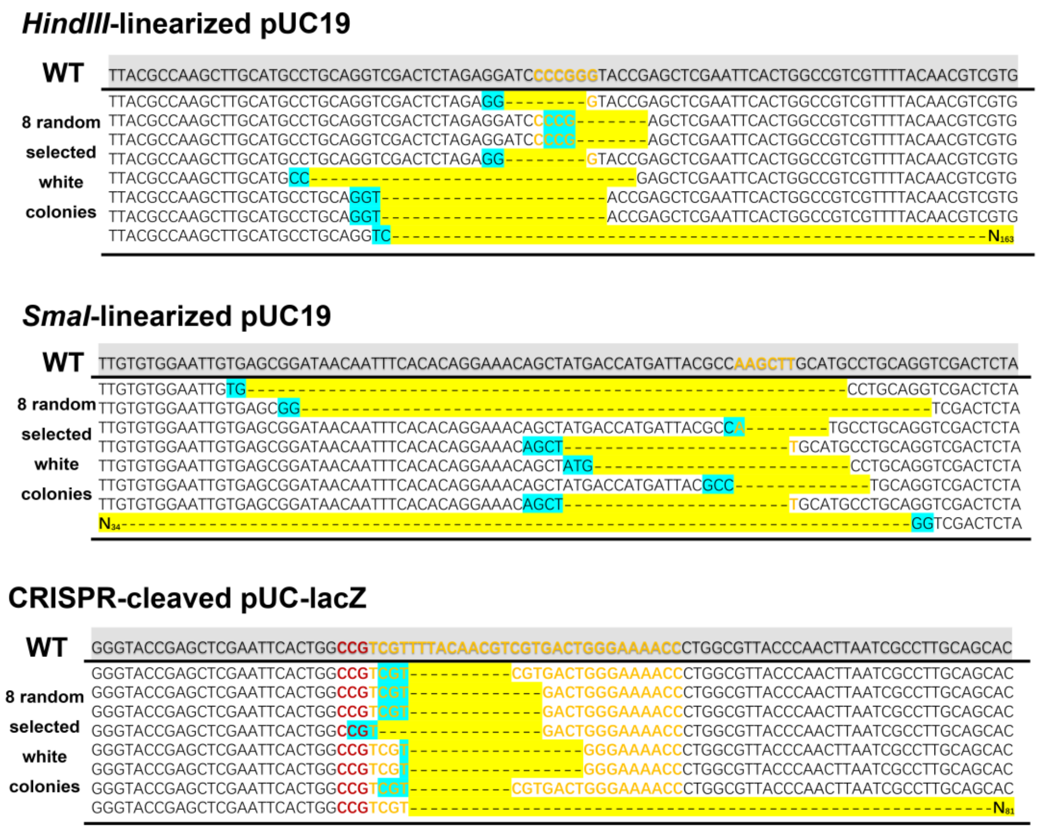

Supplementary Figure 2. Sanger sequencing analysis of 8 random selected white colonies (*lacZ*- genotype) generated by repair of *Hind*III/*Sma*I-linearized pUC19 or Cas9-cleaved pUC-lacZ, respectively. The recognition sequences of *Hind*III/*Sma*I and the LR4-CRISPR target are shown in orange. The PAM sequences are shown in red. The light blue color indicates micro-homology sequence for ending-joining and the dashed line highlighted in yellow represents the deleted nucleotides by NHEJ.

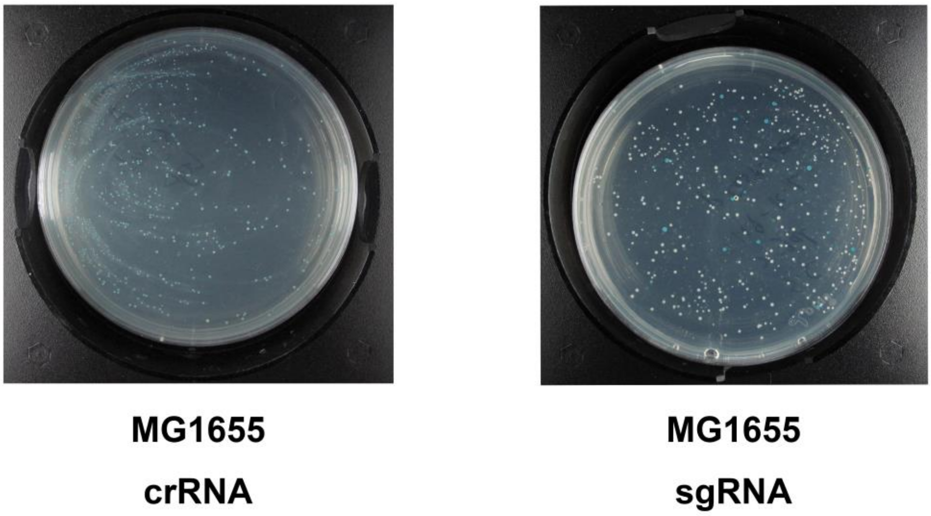

Supplementary Figure 3. Blue-white screening of the transformants to evaluate the efficiency and positivity rate of the improved CA-NHEJ system using L4 target site in *E. coli* MG1655. White colonies represent the *lacZ*$^{-}$ genotype and blue colonies represent the *lacZ*$^{+}$ genotype.

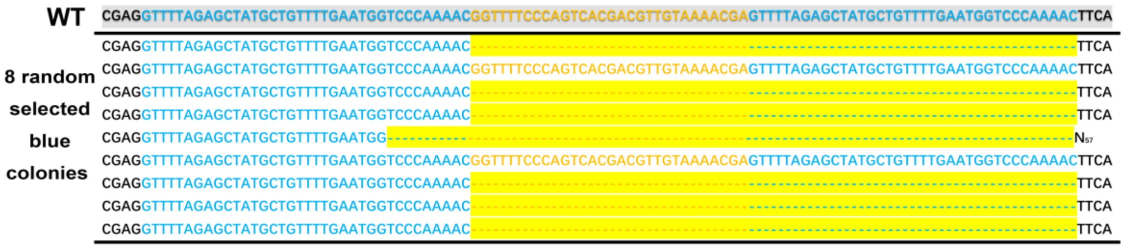

Supplementary Figure 4. Sanger sequencing analysis of the LR4 CRISPR array in 8 random selected blue colonies grown on the X-gal plate. The repeat sequences and the LR4 spacer of CRISPR array are shown in blue and orange, respectively. The dashed line highlighted in yellow indicates the deleted sequences.

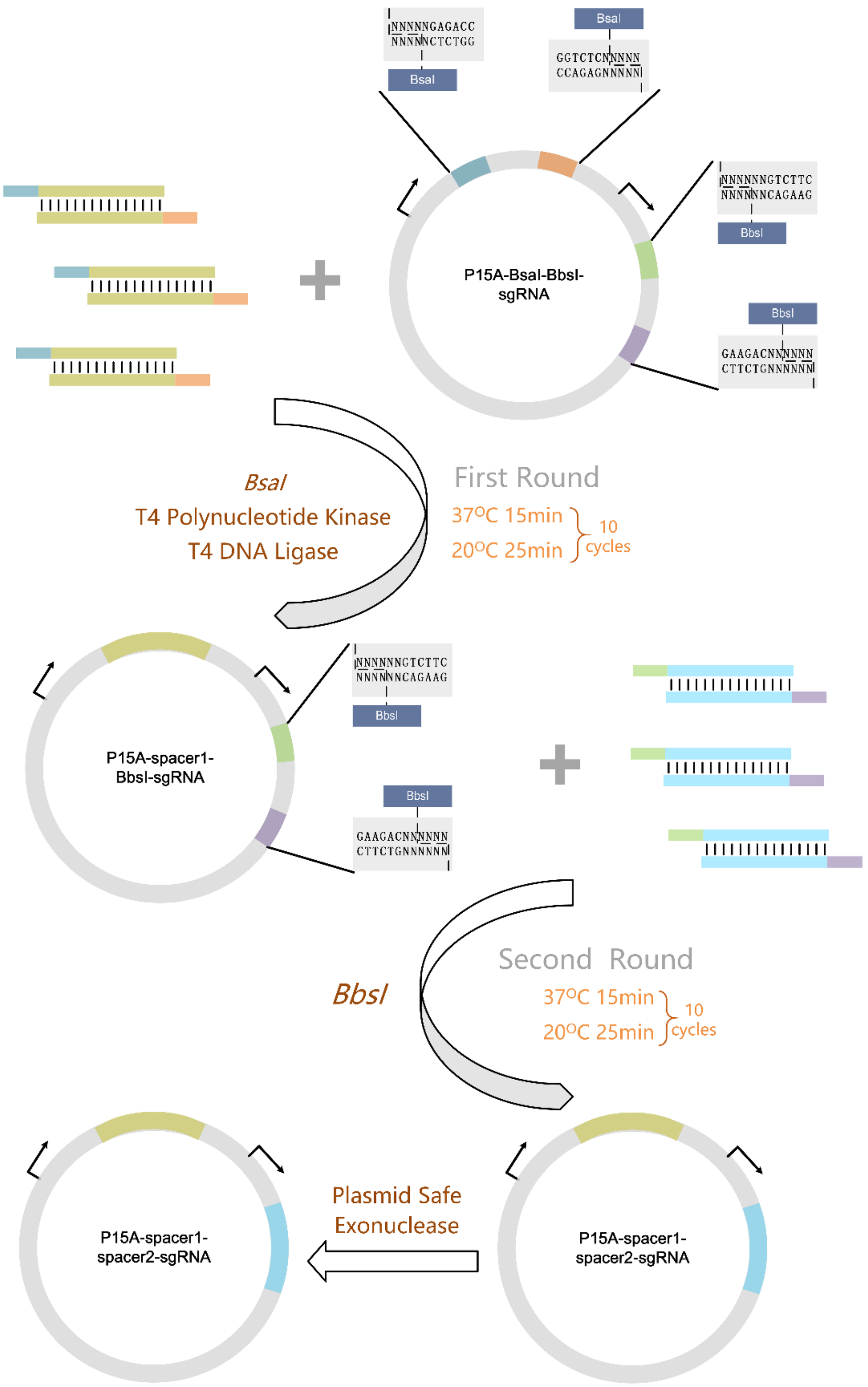

Supplementary Figure 5. One-step digestion-ligation method based on golden gate cloning to easy clone of specific sgRNA pair cassettes in plasmid p15A-BsaI-BbsI-sgRNA. The one-step digestion-ligation cloning strategy for ligation of the sgRNA pairs into the p15A-BsaI-BbsI-sgRNA plasmid was accomplished through two rounds iterative golden gate cloning in a 30-μl reaction system. Tan: spacer 1; Light blue: spacer 2.

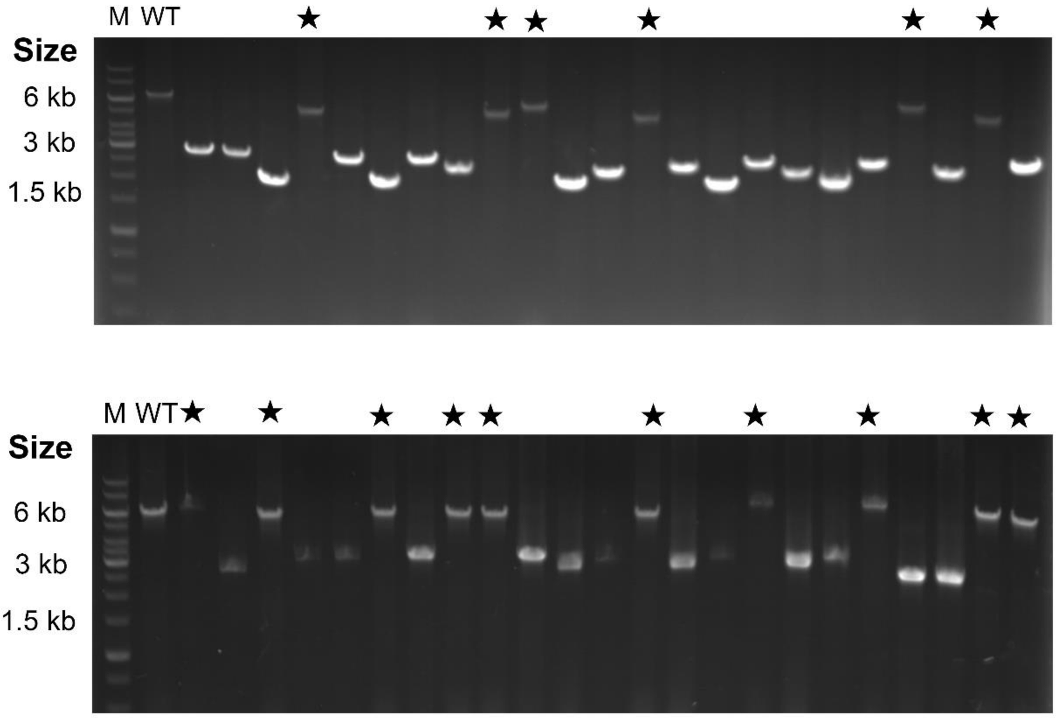

Supplementary Figure 6. Gel electrophoresis of the PCR products to distinguish the mutation types of *lacZ* by sgRNA pair L4&LR8. Asterisk indicates the frameshift mutation of *lacZ.*

Supplementary Table 1. Primers used in this study (The homologous sequences used for assembling were in bold, restriction sites were underlined).

Primers	Nucleotide sequence(5'-3')
**Primers for plasmids construction**	
lacZ-F	CCCAAAGCTTACAGCTATGACCATGATTACGGATT
lacZ-R	TCCAGATCTAGACCTTACGCGAAATACGGGCAGACAT
Cm-Ori-F	**TAGTGTGAGATCTCATCGGGCTTACTCGATGCATGCGCTAACCG**TTTTTATCAGGCTCTGGG
Cm-Ori-R	**ATCCGTACGCCTGCAGGTCTAGATTAATTAACGCCGGCGGCGGCCGC**TATGGACAGTTTTCCCTTTGATAT
cas9-F	**GCGGCCGCCGCCGGCGTTAATTAATCTAGACCTGCAGGCGTACGGAT**TACGAAATCATCCTGTGGAGC
cas9-R	**CGGTTAGCGCATGCATCGAGTAAGCCCGATGAGATCTCACACTA**CTCTTCTTTTGCCTATTATAAC
23119-ligd-F	ACCGTAAGATCTTTGACAGCTAGCTCAGTCCTAGGTATAATGCTAGCTACTAGAGAAAGAGGAGAAATACTAGATGGGTT CGGCGTCGGAGCA
23119-ligd-R	**TTAATTCATTCGCGCACCACCTCACTGG**
mku-F	**GCGGCCGGACAAGAAACCCAGTGAGGTGGTGCGCGAATGA**ATTAAAGAGGAGAATACTAGATGCGAGCCATTTGGA CGGG
mku-R	ACAATGATGCATTTAGCGCAAGAAGACAAAAATCACCTTGCGCTAATGCTCTGTTACAGTCACGGAGGCGTTGGGACG
CRISPR-F	TCCAGATCTAGATGCCTCTAGCACGCGTACCAT
CRISPR-R	TCCAGATCTAGATGACCGAATTCAACTCAACAAGT
NHEJ-F	ACCGATACTAGTTTGACAGCTCAGTCCTAGGTATAATGCTAGC
NHEJ-R	ACCGATACTAGTTTAGCGCAAGAAGACAAAAATCACCTTGCGC

Spc-F	**TCTGTTGTTTGTCGGTGAACTGGATCC**CTTAGTAAAGCCCTCGCTAGATT
Spc-R	**ACCATCATACACTAAATCAGTAAGTTGGCAGCATCACCCGACG**GCTCGTTCGCCAGCCAGGACAGA
P15A-F	**CGTCGGGTGATGCTGCCAACTTA**
P15A-R	**ACTTATATCGTATGGGGCTGACTTC**
sgRNA-F	**AGCACCTGAAGTCAGCCCCATACGATATAAGT**CTATAAAAATAGGCGTATCACGAGGC
sgRNA-R	**TTACTAAGGGATCCAGTTCACCGACAAACAACAGA**
**Primers for gene knockout**	
lac-F	CGATACCGAAGACAGCTCATGT
lac-R	TATCATCGCCGGGCTTGCCCCGT
**Primers for fragment deletion analysis**	
LacZ-JF	ATGTCCGCCGTAGCCCCTCCGATGAT
LacZ-JR	CCAGCAGGAACGGTACTTCAAAC
Lac-JF	AGCGCCTCGTCATCAATACCAAT
Lac-JR	TTCCCACAAGACAACAACCACTCC
LC-JF	CAGAACAATGAGCAGACGGAATA
LC-JR	GGAGGCTAACAGTGTCGAATAAC
MLC-JF	AGCCCTTCCATCAGAGCGACCA
MLC-JR	CACACAACCGGCACAAACCACC

Supplementary Table 2. Plasmids used in this study.

Plasmids	Relevant genotype	Reference
pUC19	Cloning vector, Amp^R	Lab stock
pCRISPR	pUC19 containing CRISPR array, kan^R	[1]
pCas9	pACYC184 containing tracr RNA, *cas9* and CRISPR array, Cm^R	[1]
pwtCas9	pUC19 containing aTc-inducible promoter $P_L$tetO-1 expressing *cas9*, Amp^R	[2]
pTKRED	Temperature-conditional replicon containing *γ, β, exo* (red recombinase), Spc^R	[3]
pCP20	Helper plasmid, Cm^R	[4]
pUC-lacZ	pUC19 containing *lacZ* gene, Amp^R	This study
pCas9 (Ts)	Temperature-conditional replicon containing *cas9*, Cm^R	This study
pCas9 (Ts)-NHEJ	pCas9 (Ts) containing p23119-*mku-ligd*, Cm^R	This study
pCas9 (Ts)-LR4	pCas9 (Ts) containing LR4-CRISPR array, Cm^R	This study
pCas9 (Ts)-NHEJ-LR4	pCas9 (Ts)-NHEJ containing LR4-CRISPR array, Cm^R	This study
pcurCas9 (Ts)-NHEJ	pCas9 (Ts)-NHEJ containing *lacI* and trc-p15A-sgRNA, Cm^R	This study
pwtCas9-NHEJ	pwtCas9 containing p23119-*mku-ligd*, Amp^R	This study
p15A-gRNA	p15A replicon containing sgRNA, spc^R	This study
p15A-BsaI-BbsI-gRNA	p15A replicon containing two sgRNA modules for one-step constructing sgRNA pairs, spc^R	This study
pCRISPR-L4	pCRISPR containing L4 CRISPR array, kan^R	This study
pCRISPR-LR4	pCRISPR containing LR4 CRISPR array, kan^R	This study
p15A-L4	p15A-gRNA containing sgRNA L4, spc^R	This study
p15A-L5	p15A-gRNA containing sgRNA L5, spc^R	This study
p15A-LR4	p15A-gRNA containing sgRNA LR4, spc^R	This study
p15A-LR6	p15A-gRNA containing sgRNA LR6, spc^R	This study

p15A-LR7	p15A-gRNA containing sgRNA LR7, spc^R	This study
p15A-LR8	p15A-gRNA containing sgRNA LR8, spc^R	This study
p15A-L4&LR8	p15A-BsaI-BbsI-gRNA containing sgRNA pair L4&LR8, spc^R	This study
p15A-LI10&LA0	p15A-BsaI-BbsI-gRNA containing sgRNA pair LI10&LA0, spc^R	This study
p15A-LI10&CR0	p15A-BsaI-BbsI-gRNA containing sgRNA pair LI10&CR0, spc^R	This study
p15A-ME17&CR0	p15A-BsaI-BbsI-gRNA containing sgRNA pair ME17&CR0, spc^R	This study

Supplementary Table 3. CRISPR target sequences used in this study.

Spacer name	Nucleotide sequence (5'-3')
L4-CRISPR	GGTTTTCCCAGTCACGACGTTGTAAAACGA
LR4-CRISPR	TCCGCCGTTTGTTCCCACGGAGAATCCGAC
L4	GTCACGACGTTGTAAAACGA
L5	GTGAGCGAGTAACAACCCGT
LR4	GTTCCCACGGAGAATCCGAC
LR6	CAACGTGACCTATCCCATTA
LR7	CCATCGCCATCTGCTGCACG
LR8	CTCCTGGAGCCCGTCAGTAT
LI10	TACGATGTCGCAGAGTATGC
LA0	ATTGGCAATAACGTCTGGAT
CR0	CCCGCCACTACTGGAGAGAA
ME17	GTTTCGCGATCGACTCGTAC

Supplementary Note 1

The sequences of core elements in this study

P_{J23119}-*ligd-mku*

ttgacagctagctcagtcctaggtataatgctagctactagagAAAGAGGAGAAATACTAGATG
GGTTCGGCGTCGGAGCAACGGGTGACGCTGACCAACGCCGACAAGG
TGCTCTATCCCGCCACCGGGACCACAAAGTCCGATATCTTCGACTACT
ACGCCGGTGTTGCCGAAGTCATGCTCGGCCACATCGCGGGACGGCCG
GCGACGCGCAAGCGCTGGCCTAACGGCGTCGACCAACCCGCGTTCTT
CGAAAAGCAGTTGGCGTTGTCGGCGCCGCCTTGGCTGTCACGTGCAA
CGGTGGCGCACCGGTCCGGGACGACGACCTATCCGATCATCGATAGC
GCAACCGGGCTGGCCTGGATCGCCCAACAGGCGGCGCTGGAGGTGC
ACGTGCCGCAGTGGCGGTTTGTCGCCGAGCCCGGATCAGGTGAGTTA
AATCCGGGCCCGGCAACGCGTTTGGTGTTCGACCTGGACCCGGGCGA
AGGCGTGATGATGGCCCAGCTGGCCGAGGTGGCGCGCGCGGTTCGTG
ATCTTCTCGCCGATATCGGGTTGGTCACCTTCCCGGTCACCAGCGGCA
GCAAGGGATTGCATCTGTACACACCGCTGGATGAGCCGGTGAGCAGC
AGGGGAGCCACGGTGTTGGCCAAGCGCGTCGCGCAGCGATTGGAGC
AGGCGATGCCCGCGTTGGTCACCTCGACCATGACCAAAAGCCTGCGG
GCCGGGAAGGTGTTTGTGGACTGGAGCCAGAACAGCGGCTCGAAGA
CCACCATCGCGCCGTACTCACTACGTGGCCGGACGCATCCGACCGTC
GCGGCGCCACGCACCTGGGCGGAGCTCGACGACCCCGCACTGCGTC
AGCTCTCCTACGACGAGGTGCTGACCCGGATTGCCCGCGACGGCGAT
CTGCTCGAGCGGCTGGATGCCGACGCTCCGGTAGCGGACCGGTTGAC
CCGATACCGCCGCATGCGCGACGCATCGAAAACTCCCGAGCCGATTCC
CACGGCGAAACCCGTTACCGGAGACGGCAATACGTTCGTCATCCAGG
AGCATCACGCGCGTCGGCCGCACTACGATTTCCGGCTGGAATGCGAC
GGCGTGCTGGTTTCGTGGGCGGTACCGAAAAACCTGCCCGACAACAC
ATCGGTTAACCATCTAGCGATACACACCGAGGACCACCCGCTGGAATA
CGCCACGTTCGAGGGCGCGATTCCCAGCGGGGAGTACGGCGCCGGCA
AGGTGATCATCTGGGACTCCGGCACTTACGACACCGAGAAGTTCCAC
GATGACCCGCACACGGGGGAGGTCATCGTGAATCTGCACGGCGGCCG

GATCTCTGGGCGTTATGCGCTGATTCGGACCAACGGCGATCGGTGGCT
GGCGCACCGCCTAAAGAATCAGAAAGACCAGAAGGTGTTCGAGTTCG
ACAATCTGGCCCCAATGCTTGCCACGCACGGCACGGTGGCCGGTCTA
AAGGCCAGCCAGTGGGCGTTCGAAGGCAAGTGGGACGGCTACCGGTT
GCTGGTTGAGGCTGACCACGGCGCCGTGCGGCTGCGGTCCCGCAGC
GGGCGCGATGTCACCGCCGAGTATCCGCAATTGCGGGCATTGGCGGA
GGATCTCGCCGATCACCACGTGGTGCTGGACGGCGAGGCCGTCGTAC
TTGACTCCTCTGGTGTGCCCAGCTTCAGCCAGATGCAGAATCGGGGC
CGCGACACCCGTGTCGAGTTCTGGGCGTTCGACCTGCTCTACCTCGA
CGGCCGCGCGCTGCTAGGCACCCGCTACCAAGACCGGCGTAAGCTGC
TCGAAACCCTAGCTAACGCAACCAGTCTCACCGTTCCCGAGCTGCTGC
CCGGTGACGGCGCCCAAGCGTTTGCGTGCTCGCGCAAGCACGGCTGG
GAGGGCGTGATCGCCAAGAGGCGTGACTCGCGCTATCAGCCGGGCCG
GCGCTGCGCGTCGTGGGTCAAGGACAAGCACTGGAACACCCAGGAA
GTCGTCATTGGTGGCTGGCGCGCCGGGGAAGGCGGGCGCAGCAGTG
GCGTCGGGTCGCTGCTCATGGGCATCCCCGGTCCAGGTGGGCTGCAG
TTCGCCGGGCGGGTCGGTACCGGCCTCAGCGAACGCGAACTGGCCAA
CCTCAAGGAGATGCTGGCGCCGCTGCATACCGACGAGTCCCCCTTCG
ACGTACCACTGCCCGCGCGTGACGCCAAGGGCATCACATATGTCAAGC
CGGCGCTGGTTGCAGAGGTGCGCTACAGCGAGTGGACTCCGGAGGG
CCGGCTGCGTCAATCAAGCTGGCGTGGGCTGCGGCCGGACAAGAAAC
CCAGTGAGGTGGTGCGCGAATGAtactagagATTAAAGAGGAGAATACTA
GATGCGAGCCATTTGGACGGGTTCGATCGCCTTCGGGCTGGTGAACG
TGCCGGTCAAGGTGTACAGCGCTACCGCAGACCACGACATCAGGTTC
CACCAGGTGCACGCCAAGGACAACGGACGCATCCGGTACAAGCGCGT
CTGCGAGGCGTGTGGCGAGGTGGTCGACTACCGCGATCTTGCCCGGG
CCTACGAGTCCGGCGACGGCCAAATGGTGGCGATCACCGACGACGAC
ATCGCCAGCTTGCCTGAAGAACGCAGCCGGGAGATCGAGGTGTTGGA
GTTCGTCCCCGCCGCCGACGTGGACCCGATGATGTTCGACCGCAGCT
ACTTTTTGGAGCCTGATTCGAAGTCGTCGAAATCGTATGTGCTGCTGG
CTAAGACACTCGCCGAAACCGACCGGATGGCGATCGTGCATTTCACGC
TGCGCAACAAGACCAGGCTGGCGGCGTTGCGCGTCAAGGATTTCGGC
AAGCGAGAGGTGATGATGGTGCACACGTTGCTGTGGCCCGATGAGAT
CCGCGACCCCGACTTCCCGGTGCTGGACCAGAAGGTGGAGATCAAAC

CCGCGGAACTCAAGATGGCCGGCCAGGTGGTGGACTCGATGGCCGAC
GACTTCAATCCGGACCGCTACCACGACACCTACCAGGAGCAGTTACAG
GAGCTGATCGACACCAAACTCGAAGGTGGGCAGGCATTTACCGCCGA
GGACCAACCGAGGTTGCTGGACGAGCCCGAAGACGTCTCCGACCTGC
TCGCCAAGCTGGAGGCCAGCGTGAAGGCGCGCTCGAAGGCCAACTCA
AACGTCCCAACGCCTCCGTGA

tracRNA-Cas9-CRISPR array

AAAAAAAGCACCGACTCGGTGCCACTTTTTCAAGTTGATAACGGACTA
GCCTTATTTTAACTTGCTATGCTGTTTTGAATGGTTCCAACAAGATTAT
TTTATAACTTTTATAACAAATAATCAAGGAGAAATTCAAAGAAATTTATC
AGCCATAAAACAATACTTAATACTATAGAATGATAACAAAATAAACTACT
TTTTAAAAGAATTTTGTGTTATAATCTATTTATTATTAAGTATTGGGTAAT
ATTTTTTGAAGAGATATTTTGAAAAAGAAAAATTAAAGCATATTAAACT
AATTTCGGAGGTCATTAAAACTATTATTGAAATCATCAAACTCATTATG
GATTTAATTTAAACTTTTTATTTTAGGAGGCAAAAATGGATAAGAAATA
CTCAATAGGCTTAGATATCGGCACAAATAGCGTCGGATGGGCGGTGAT
CACTGATGAATATAAGGTTCCGTCTAAAAAGTTCAAGGTTCTGGGAAA
TACAGACCGCCACAGTATCAAAAAAAATCTTATAGGGGCTCTTTTATTT
GACAGTGGAGAGACAGCGGAAGCGACTCGTCTCAAACGGACAGCTCG
TAGAAGGTATACACGTCGGAAGAATCGTATTTGTTATCTACAGGAGATT
TTTTCAAATGAGATGGCGAAAGTAGATGATAGTTTCTTTCATCGACTTG
AAGAGTCTTTTTTGGTGGAAGAAGACAAGAAGCATGAACGTCATCCTA
TTTTTGGAAATATAGTAGATGAAGTTGCTTATCATGAGAAATATCCAAC
TATCTATCATCTGCGAAAAAAATTGGTAGATTCTACTGATAAAGCGGAT
TTGCGCTTAATCTATTTGGCCTTAGCGCATATGATTAAGTTTCGTGGTC
ATTTTTTGATTGAGGGAGATTTAAATCCTGATAATAGTGATGTGGACAA
ACTATTTATCCAGTTGGTACAAACCTACAATCAATTATTTGAAGAAAAC
CCTATTAACGCAAGTGGAGTAGATGCTAAAGCGATTCTTTCTGCACGA
TTGAGTAAATCAAGACGATTAGAAAATCTCATTGCTCAGCTCCCCGGT
GAGAAGAAAAATGGCTTATTTGGGAATCTCATTGCTTTGTCATTGGGT
TTGACCCCTAATTTTAAATCAAATTTTGATTTGGCAGAAGATGCTAAAT
TACAGCTTTCAAAAGATACTTACGATGATGATTTAGATAATTTATTGGC
GCAAATTGGAGATCAATATGCTGATTTGTTTTTGGCAGCTAAGAATTTA

TCAGATGCTATTTTACTTTCAGATATCCTAAGAGTAAATACTGAAATAAC
TAAGGCTCCCCTATCAGCTTCAATGATTAAACGCTACGATGAACATCAT
CAAGACTTGACTCTTTTAAAAGCTTTAGTTCGACAACAACTTCCAGAA
AAGTATAAAGAAATCTTTTTTGATCAATCAAAAAACGGATATGCAGGTT
ATATTGATGGGGGAGCTAGCCAAGAAGAATTTTATAAATTTATCAAACC
AATTTTAGAAAAAATGGATGGTACTGAGGAATTATTGGTGAAACTAAAT
CGTGAAGATTTGCTGCGCAAGCAACGGACCTTTGACAACGGCTCTATT
CCCCATCAAATTCACTTGGGTGAGCTGCATGCTATTTTGAGAAGACAA
GAAGACTTTTATCCATTTTTAAAAGACAATCGTGAGAAGATTGAAAAA
ATCTTGACTTTTCGAATTCCTTATTATGTTGGTCCATTGGCGCGTGGCA
ATAGTCGTTTTGCATGGATGACTCGGAAGTCTGAAGAAACAATTACCC
CATGGAATTTTGAAGAAGTTGTCGATAAAGGTGCTTCAGCTCAATCAT
TTATTGAACGCATGACAAACTTTGATAAAAATCTTCCAAATGAAAAAGT
ACTACCAAAACATAGTTTGCTTTATGAGTATTTTACGGTTTATAACGAAT
TGACAAAGGTCAAATATGTTACTGAAGGAATGCGAAAACCAGCATTTC
TTTCAGGTGAACAGAAGAAAGCCATTGTTGATTTACTCTTCAAAACAA
ATCGAAAAGTAACCGTTAAGCAATTAAAAGAAGATTATTTCAAAAAAAT
AGAATGTTTTGATAGTGTTGAAATTTCAGGAGTTGAAGATAGATTTAAT
GCTTCATTAGGTACCTACCATGATTTGCTAAAAATTATTAAAGATAAAG
ATTTTTTGGATAATGAAGAAAATGAAGATATCTTAGAGGATATTGTTTTA
ACATTGACCTTATTTGAAGATAGGGAGATGATTGAGGAAAGACTTAAA
ACATATGCTCACCTCTTTGATGATAAGGTGATGAAACAGCTTAAACGTC
GCCGTTATACTGGTTGGGGACGTTTGTCTCGAAAATTGATTAATGGTAT
TAGGGATAAGCAATCTGGCAAAACAATATTAGATTTTTTGAAATCAGAT
GGTTTTGCCAATCGCAATTTTATGCAGCTGATCCATGATGATAGTTTGA
CATTTAAAGAAGACATTCAAAAAGCACAAGTGTCTGGACAAGGCGATA
GTTTACATGAACATATTGCAAATTTAGCTGGTAGCCCTGCTATTAAAAA
AGGTATTTTACAGACTGTAAAAGTTGTTGATGAATTGGTCAAAGTAATG
GGGCGGCATAAGCCAGAAAATATCGTTATTGAAATGGCACGTGAAAAT
CAGACAACTCAAAAGGGCCAGAAAAATTCGCGAGAGCGTATGAAACG
AATCGAAGAAGGTATCAAAGAATTAGGAAGTCAGATTCTTAAAGAGCA
TCCTGTTGAAAATACTCAATTGCAAAATGAAAAGCTCTATCTCTATTAT
CTCCAAAATGGAAGAGACATGTATGTGGACCAAGAATTAGATATTAATC
GTTTAAGTGATTATGATGTCGATCACATTGTTCCACAAAGTTTCCTTAA

AGACGATTCAATAGACAATAAGGTCTTAACGCGTTCTGATAAAAATCGT
GGTAAATCGGATAACGTTCCAAGTGAAGAAGTAGTCAAAAAGATGAAA
AACTATTGGAGACAACTTCTAAACGCCAAGTTAATCACTCAACGTAAG
TTTGATAATTTAACGAAAGCTGAACGTGGAGGTTTGAGTGAACTTGAT
AAAGCTGGTTTTATCAAACGCCAATTGGTTGAAACTCGCCAAATCACT
AAGCATGTGGCACAAATTTTGGATAGTCGCATGAATACTAAATACGATG
AAAATGATAAACTTATTCGAGAGGTTAAAGTGATTACCTTAAAATCTAA
ATTAGTTTCTGACTTCCGAAAAGATTTCCAATTCTATAAAGTACGTGAG
ATTAACAATTACCATCATGCCCATGATGCGTATCTAAATGCCGTCGTTG
GAACTGCTTTGATTAAGAAATATCCAAAACTTGAATCGGAGTTTGTCTA
TGGTGATTATAAAGTTTATGATGTTCGTAAAATGATTGCTAAGTCTGAG
CAAGAAATAGGCAAAGCAACCGCAAAATATTTCTTTTACTCTAATATCA
TGAACTTCTTCAAAACAGAAATTACACTTGCAAATGGAGAGATTCGCA
AACGCCCTCTAATCGAAACTAATGGGGAAACTGGAGAAATTGTCTGGG
ATAAAGGGCGAGATTTTGCCACAGTGCGCAAAGTATTGTCCATGCCCC
AAGTCAATATTGTCAAGAAAACAGAAGTACAGACAGGCGGATTCTCCA
AGGAGTCAATTTTACCAAAAAGAAATTCGGACAAGCTTATTGCTCGTA
AAAAAGACTGGGATCCAAAAAAATATGGTGGTTTTGATAGTCCAACGG
TAGCTTATTCAGTCCTAGTGGTTGCTAAGGTGGAAAAAGGGAAATCGA
AGAAGTTAAAATCCGTTAAAGAGTTACTAGGGATCACAATTATGGAAA
GAAGTTCCTTTGAAAAAAATCCGATTGACTTTTTAGAAGCTAAAGGAT
ATAAGGAAGTTAAAAAAGACTTAATCATTAAACTACCTAAATATAGTCTT
TTTGAGTTAGAAAACGGTCGTAAACGGATGCTGGCTAGTGCCGGAGA
ATTACAAAAAGGAAATGAGCTGGCTCTGCCAAGCAAATATGTGAATTT
TTTATATTTAGCTAGTCATTATGAAAAGTTGAAGGGTAGTCCAGAAGAT
AACGAACAAAAACAATTGTTTGTGGAGCAGCATAAGCATTATTTAGAT
GAGATTATTGAGCAAATCAGTGAATTTTCTAAGCGTGTTATTTTAGCAG
ATGCCAATTTAGATAAAGTTCTTAGTGCATATAACAAACATAGAGACAA
ACCAATACGTGAACAAGCAGAAAATATTATTCATTTATTTACGTTGACG
AATCTTGGAGCTCCCGCTGCTTTTAAATATTTTGATACAACAATTGATC
GTAAACGATATACGTCTACAAAAGAAGTTTTAGATGCCACTCTTATCCA
TCAATCCATCACTGGTCTTTATGAAACACGCATTGATTTGAGTCAGCTA
GGAGGTGACTGAAGTATATTTTAGATGAAGATTATTTCTTAATAACTAA
AAATATGGTATAATACTCTTAATAAATGCAGTAATACAGGGGCTTTTCAA

GACTGAAGTCTAGCTGAGACAAATAGTGCGATTACGAAATTTTTTAGA
CAAAAATAGTCTACGAGGTTTTAGAGCTATGCTGTTTTGAATGGTCCCA
AAACTGAGACCAGTCTCGGAAGCTCAAAGGTCTCGTTTTAGAGCTATG
CTGTTTTGAATGGTCCCAAAAC

P_{J23119}-*BsaI*-sgRNA

**TTGACAGCTAGCTCAGTCCTAGGTATAATACTAGTTGAGACCAGTCTC
GGAAGCTCAAAGGTCTCGGTTTTAGAGCTAGAAATAGCAAGTTAAAAT
AAGGCTAGTCCGTTATCAACTTGAAAAAGTGGCACCGAGTCGGTGC**

1 Jiang, W. Y., Bikard, D., Cox, D., Zhang, F. & Marraffini, L. A. RNA-guided editing of bacterial genomes using CRISPR-Cas systems. *Nat Biotechnol* **31**, 233-239, doi:10.1038/nbt.2508 (2013).

2 Qi, L. S. *et al.* Repurposing CRISPR as an RNA-guided platform for sequence-specific control of gene expression. *Cell* **152**, 1173-1183, doi:10.1016/j.cell.2013.02.022 (2013).

3 Kuhlman, T. E. & Cox, E. C. Site-specific chromosomal integration of large synthetic constructs. *Nucleic Acids Res* **38**, doi:ARTN e9210.1093/nar/gkp1193 (2010).

4 Cherepanov, P. P. & Wackernagel, W. Gene disruption in *Escherichia coli*: TcR and KmR cassettes with the option of Flp-catalyzed excision of the antibiotic-resistance determinant. *Gene* **158**, 9-14 (1995).
